# Comments on “Validity and Reliability of the Flourishing Scale in a Sample of Older Adults in Iran” [Letter]

**DOI:** 10.2147/CIA.S268539

**Published:** 2020-07-09

**Authors:** I Ketut Andika Priastana

**Affiliations:** 1 Universitas Triatma Mulya, Badung, Bali, Indonesia

## Dear editor

I have read the study of Fassih-Ramandi et al titled “Validity and Reliability of the Flourishing Scale in a Sample of Older Adults in Iran” with great interest. In this paper, the authors reported their study on Persian validation of the Flourishing Scale and reported it as a reliable tool for the assessment of psychological well-being in older adults.^1^

First, I found a discrepancy between citation and bibliography writing, namely citation in Diener et al. The author needs to explain that there are 2 publications conducted by Diener et al related to this research in 2009 and 2010. The first publication in 2009, Diener et al wrote using the term Psychological Well-Being for assessing well-being.^2^ In 2010, Diener et al changed its name to the Flourishing Scale.^3^

In selecting the comparison instrument, it is also necessary to explain how the authors chose the Oxford happiness questionnaire and the feasibility of this instrument to be compared with the Flourishing Scale.

Another point is the authors explain the present study employed the use of the Persian version of the FS that was translated by Moradi Siah Afshadi et al.^4^ In the discussion of Construct Validity Assessment is writing the Persian version of the Flourishing Scale while the table described is the original version. This is very important so that readers understand the results of this study, whether the tested is the Persian version or the original version.
